# Glucagon-Like Peptide-1 Secretory Function as an Independent Determinant of Blood Pressure: Analysis in the Tanno-Sobetsu Study

**DOI:** 10.1371/journal.pone.0067578

**Published:** 2013-07-02

**Authors:** Mayumi Yoshihara, Hiroshi Akasaka, Hirofumi Ohnishi, Takayuki Miki, Tetsuaki Furukawa, Satoshi Yuda, Shigeyuki Saitoh, Tetsuji Miura

**Affiliations:** 1 Department of Cardiovascular, Renal and Metabolic Medicine, Sapporo Medical University School of Medicine, Sapporo, Japan; 2 Department of Public Health, Sapporo Medical University School of Medicine, Sapporo, Japan; 3 Department of Clinical Laboratory Medicine, Sapporo Medical University School of Medicine, Sapporo, Japan; 4 Department of Nursing, Sapporo Medical University School of Health Sciences, Sapporo, Japan; INRCA, Italy

## Abstract

**Aims:**

Roles of glucagon-like peptide-1 (GLP-1) in extra-pancreatic tissues remain unclear. The aim of this study was to examine determinants of GLP-1 secretory function and possible contribution of GLP-1 to blood pressure (BP) regulation.

**Methods and Results:**

We recruited 128 subjects who received annual examinations and 75g-oral glucose tolerance tests (OGTT) in the Tanno-Sobetsu cohort. Subjects on regular medications for cardiovascular and/or metabolic diseases were excluded, and data for the remaining 103 subjects were used for the univariate and multivariate analyses. Age, plasma glucose (PG), hemoglobin A1c (HbA1c), plasma insulin, and serum lipids were not selected as independent determinants of fasting GLP-1 level by multiple linear regression analysis. However, age and female sex were selected as independent positive determinants of the area under the curve of GLP-1 level during OGTT (AUC_GLP-1_), an index of GLP-1 secretory function. Multiple linear regression analysis indicated that AUC_GLP-1_ was an independent negative predictor of systolic BP (SBP), while AUC_GLP-1_ was not correlated with fasting PG or HbA1c level. In subgroup analyses using the median of AUC_GLP-1_ to divide the study subjects into high and low GLP-1 response groups, AUC_GLP-1_ was significantly correlated with both SBP and diastolic BP (r = 0.40 and 0.28, respectively) in the low GLP-1 response group but not in the high GLP-1 response group.

**Conclusions:**

The results of the present study suggest that GLP-1 secretory function is involved in prevention of BP elevation and that the GLP-1 response to oral glucose rather increases with aging perhaps as an adaptive phenomenon.

## Introduction

Glucagon-like peptide 1 (GLP-1), one of the incretins, is secreted from L-cells in the small intestine after meals, contributing to enhancement of post-prandial insulin secretion, suppression of glucagon secretion and deceleration of gastric emptying [1], [2]. Both increase in vagal tone and activation of L-cells by dietary nutrients participate in triggering GLP-1 secretion into the blood stream. GLP-1 is rapidly inactivated by dipeptidyl peptidase-4 (DPP-4) and is eliminated mainly from the kidney. Post-prandial level of GLP-1 is reduced in patients with type 2 diabetes [3], [4], and thus DPP-4 inhibitors and GLP-1 analogues have been widely used for control of plasma glucose (PG) levels in diabetic patients. However, the physiological functions of GLP-1 in extra-pancreatic tissues have not been fully characterized, though regulation of bone metabolism, progenitor cell proliferation in the brain, lipogenesis in adipose tissue and angiogenesis in the heart have been proposed [1], [2]. It is also unclear how basal (i.e., pre-prandial) GLP-1 level is regulated and how GLP-1 secretory capacity is regulated in healthy subjects.

In the present study, we first examined whether basal (fasting) GLP-1 level and GLP-1 secretory function are determined by any of the demographic or metabolic parameters in apparently healthy subjects who participated in annual health examinations. Second, we examined the possibility that GLP-1 secretory capacity is involved in blood pressure (BP) regulation. The rationale for this hypothesis is two-fold. First, earlier studies [5], [6] have demonstrated that GLP-1 enhances urinary sodium excretion. Second, GLP-1 and its analogues lowered BP in Dahl salt–sensitive rats [7] and in patients with type 2 diabetes [8]–[11]. Apparently healthy subjects in the Tanno-Sobetsu cohort [12], [13] were recruited to the present study, and we examined relationships between fasting plasma GLP-1 level, plasma GLP-1 response to oral glucose loading, and demographic and metabolic parameters. Results of the analysis suggested that endogenous GLP-1 plays a role in BP regulation and that GLP-1 response to glucose loading rather increases with aging perhaps as an adaptive response.

## Methods

The protocol of this study was approved by the Ethics Committee of Sapporo Medical University and we conducted this study according to the principles expressed in the Declaration of Helsink. Written informed consent was obtained from all subjects who participated in the Tanno-Sobetsu Study [12], [13].

### Study Subjects

We recruited participants in the Tanno-Sobetsu Study [12], [13], a study with a population-based prospective cohort design, to the present analyses. In the Tanno-Sobetsu Study, residents of two towns, Tanno and Sobetsu, in Japan were recruited for annual or biannual medical examination, including standard blood and urine tests and electrocardiogram. Medical history, including use of medications, was taken and recorded by registered nurses. Parts of plasma samples were frozen and stored for later analyses. If the annual examination showed that fasting PG was 100 ∼ 125 (mg/dl) and/or glycohemoglobin A1c (HbA1c in national glycohemoglobin standardization program [NGSP] scale) was 5.6 ∼ 6.4%, the subject was invited to undergo an oral glucose tolerance test (OGTT) scheduled one month after the annual examination. From 2009 ∼ 2011, 881 subjects received annual examinations in Sobetsu and 315 of them received invitation to OGTT. Totally 128 subjects underwent OGTTs and were recruited to the present study. They did not differ from subjects who did not respond to invitation to OGTT (n = 187) in demographic parameters (i.e., age, sex, BP, fasting PG, HbA1c, serum lipids and renal function indices) (data not shown). Based on history taken by the nurses, 25 of 128 subjects were excluded due to clinical diagnosis of diabetes mellitus and/or regular medications for cardiovascular or metabolic diseases. The remaining 103 subjects constituted the study population for the present analyses.

### Measurements

Medical examinations were performed in the early morning after an overnight fast. In physical examinations, systolic BP (SBP) and diastolic BP (DBP) were measured twice after a 5-min rest on a seat and the values were averaged. Body mass index (BMI, kg/m^2^) was calculated as weight (kg)/height^2^ (meters). Urine was sampled for determination of albumin and creatinine (Cr) levels. Peripheral venous blood was drawn for determination of high-density lipoprotein cholesterol (HDL-C, mg/dL), total cholesterol (TCHO, mg/dL), fasting PG, triglyceride (TG), serum Cr, high-sensitivity C-reactive protein (hs-CRP) and brain natriuretic peptide (BNP). Low-density lipoprotein cholesterol (LDL-C, mg/dL) was calculated by the Friedewald formula (TCHO - HDL - TG/5). Estimated glomerular filtration rate (eGFR) was calculated from data on serum Cr, age and sex by use of equations for Japanese [14]. HbA1c was determined by using latex coagulation method and expressed in NGSP scale. OGTT was performed, and PG, immunoreactive insulin (IRI) and GLP-1 were measured before and at 60 and 120 min after drinking Trelan-G™ (75 g glucose in 225 ml water). PG and IRI levels were measured by the hexokinase method and enzyme immunoassay, respectively. Blood samples for GLP-1 assay were collected in tubes containing DPP-4 inhibitor (BD P700, Becton, Dickinson and Co.). Plasma intact GLP-1 level was determined by an ELISA kit (GLP-1 Active ELISA kit, EGLP-35k, Millipore Inc.), which measures intact GLP-1 without cross-reacting with the inactive form of GLP-1 (9–36). As indices of insulin sensitivity, homeostasis model assessment of insulin resistance (HOMA-IR) and Matsuda-DeFronzo index were calculated as previously reported [15], [16].

### Statistical Analysis

Numeric variables are expressed as means ± SD. Analysis of variance was used for testing significant differences between group means. As an index of GLP-1 secretory function, area under the curve (AUC) of GLP-1 in the OGTT (AUC_GLP-1_) was calculated by use of the trapezoidal rule. Similarly, AUCs of PG (AUC_PG_) and IRI (AUC_IRI_) in the OGTT were also calculated. Relationships between parameters were examined by use of simple and multiple linear regression analyses. In multiple linear regression analysis, we prepared several models by using all or different combinations of parameters as independent variables for calculation of both regression coefficients and Akaike Information Criterion (AIC). Among the candidate models, we selected the best-fit model using Akaike's Information Criterion (AIC) for each dependent variable. Differences in time courses of PG and IRI in the OGTT were tested by two-way repeated measures analysis of variance and Bonferroni *post hoc test* for multiple comparisons. Statistical analyses were carried out using JMP (version7 SAS Institute, Cary, NC, USA). Difference was considered to be statistically significant if p was less than 0.05.

## Results

### Demographic Characteristics of Study Subjects

Demographic and clinical parameters in the study subjects are shown in Table 1. Women had lower SBP and DBP, higher HDL-C and lower TG level than those in men. While fasting PG and fasting GLP-1 were comparable in men and women, AUC_PG_ was smaller and AUC_GLP-1_ tended to be higher in women than in men. The other parameters were comparable in men and women.

**Table 1:** Demographic and clinical parameters in study subjects.

	All (n = 103)	Men (n = 43)	Women (n = 60)
Age (years)	65±9	67±8	64±10
BMI (kg/m^2^)	24.0±3.8	24.5±2.8	23.7±4.4
SBP (mmHg)	140±20	146±21	135±17*
DBP (mmHg)	79±11	83±10	76±11*
Fasting PG (mg/dl)	96.6±9.7	97.6±9.1	95.8±10.2
AUC_PG_ (a.u.)	1647±335	1773±323	1557±311*
Fasting IRI (µIU/ml)	6.2±4.6	6.9±5.7	5.7±3.5
AUC_IRI_ (a.u.)	579±397	629±447	543±337
Fasting GLP-1 (pmol/l)	3.33±1.81	3.27±1.76	3.37±1.87
AUC_GLP-1_ (a.u.)	892±544	725±465	1012±568
LDL-C (mg/dl)	131±30	130±31	131±29
HDL-C (mg/dl)	66±22	58±16	71±25*
Triglyceride (mg/dl)	118±62	132±74	107±50*
HbA1c (%)	5.7±0.2	5.7±0.2	5.7±0.2
S-Cr (mg/dl)	0.71±0.14	0.81±0.14	0.63±0.09*
eGFR (mL/min/1.73 m^2^)	72.2±12.5	72.7±13.9	71.9±11.5
U-Alb/U-Cr (mg/gCr)	17.0±54.2	25.8±84.3	11.0±8.6
BNP (pg/ml)	20.9±16.4	17.6±15.9	23.3±16.5
hs-CRP (mg/dl)	0.08±0.10	0.10±0.12	0.07±0.09
Matsuda-DeFronzo index	8.1±4.6	7.5±4.9	8.5±4.3
HOMA-IR	1.5±1.2	1.7±1.5	1.4±0.9
HOMA-β	67.5±43.3	71.4±49.6	64.7±38.3

Data are presented as means ± SD.

* p<0.05 vs. Men.

SBP, systolic blood pressure; DBP, diastolic blood pressure; PG, plasma glucose; IRI, immunoreactive insulin; AUC_GLP-1_, area under the curve of GLP-1 level during 75 g oral glucose tolerance test; S-Cr, serum creatinine; U-Alb/U-Cr, urinary albumin concentration-to-urinary creatinine concentration ratio; hs-CRP, high-sensitivity C-reactive protein; a.u., arbitrary unit.

Of the 103 subjects, 52 subjects (50.5%) had SBP>140 mmHg and/or DBP>90 mmHg. In the OGTT, impaired glucose tolerance, impaired fasting PG, and diabetic pattern were observed in 33, 2 and 2 subjects, respectively. HbA1c levels in the two subjects showing a diabetic pattern in the OGTT were 5.7% and 6.1%.

### Relationships between GLP-1 Secretory Function and Metabolic Parameters

We first examined whether basal (fasting) GLP-1 level correlates with age, SBP, DBP, fasting plasma IRI, fasting PG, serum lipids, HbA1c, HOMA-IR or Matsuda-DeFronzo index. However, none of the parameters were correlated with basal GLP-1 (Table 2) or selected as an independent predictor of basal GLP-1 in multiple linear regression analysis (data not shown).

**Table 2:** Univariate linear regression analyses for fasting GLP-1 level and AUC_GLP-1_.

	Fasting GLP-1 (pmol/L)		AUC_GLP-1_	
	r	p	r	p
Age (years)	−0.068	0.49	0.18	0.073
BMI (kg/m^2^)	−0.13	0.20	−0.23	0.017
SBP (mmHg)	−0.15	0.13	−0.26	0.0085
DBP (mmHg)	0.029	0.77	−0.15	0.13
Fasting PG (mg/dl)	−0.025	0.80	−0.049	0.62
Fasting IRI (µIU/ml)	0.024	0.81	−0.12	0.22
LDL-C (mg/dl)	−0.082	0.41	−0.14	0.15
HDL-C (mg/dl)	−0.036	0.71	0.16	0.12
Triglyceride (mg/dl)	−0.027	0.79	−0.042	0.68
HbA1c (%)	0.12	0.24	0.15	0.13
S-Cr (mg/dl)	−0.092	0.36	−0.091	0.36
eGFR (ml/min/1.73 m^2^)	0.096	0.34	−0.16	0.11
U-Alb/U-Cr (mg/gCr)	−0.076	0.45	−0.13	0.21
BNP (pg/ml)	−0.17	0.087	0.14	0.17
hs-CRP (mg/dl)	0.020	0.95	−0.041	0.68
Matsuda-DeFronzo index	−0.077	0.44	0.020	0.84
HOMA-IR	0.020	0.84	−0.12	0.23
HOMA-β	0.018	0.86	−0.12	0.24

SBP, systolic blood pressure; DBP, diastolic blood pressure; PG, plasma glucose; IRI, immunoreactive insulin; AUC_GLP-1_, area under the curve of GLP-1 level during 75 g oral glucose tolerance test; S-Cr, serum creatinine; U-Alb/U-Cr, urinary albumin concentration-to-urinary creatinine concentration ratio; hs-CRP, high-sensitivity C-reactive protein; a.u., arbitrary unit.

In univariate linear regression analysis, AUC_GLP-1_, an index of secretory function of GLP-1, correlated with BMI and SBP and tended to correlate with age (Table 2). However, in multiple linear regression analysis, sex, age and SBP were selected as independent determinants as shown in Table 3. No significant association was detected between AUC_GLP-1_ and HbA1c, fasting TG or cholesterol levels or eGFR, and addition of any of these parameters to age, sex and SBP did not improve prediction of AUC_GLP-1_. AUC_GLP-1_ was negatively correlated with AUC_PG_ (r = −0.28, p = 0.004) but not with AUC_IRI_ (r = 0.013, p = 0.896).

**Table 3:** Multiple linear regression analysis for AUC_GLP-1_ and independent variables.

	B	SE	β	t	p
Sex	118.0	53.1	0.215	2.22	0.029
Age (years)	12.82	6.18	0.217	2.08	0.041
BMI (kg/m^2^)	−21.39	13.4	−0.150	−1.60	0.113
SBP (mmHg)	−6.221	2.66	−0.226	−2.34	0.022
eGFR (ml/min/1.73 m^2^)	−1.814	4.36	−0.042	−0.42	0.679

B, partial regression coefficient; SE, standard error; β, standardized partial regression coefficient.

The square of the coefficient of multiple correlation (R^2^) in this model = 0.19.

In multiple regression analyses for fasting PG level and for HbA1c level, neither fasting GLP-1 nor AUC_GLP-1_ was selected as a significant determinant (data not shown).

### Relationship between BP and GLP-1

**Figure 1:**
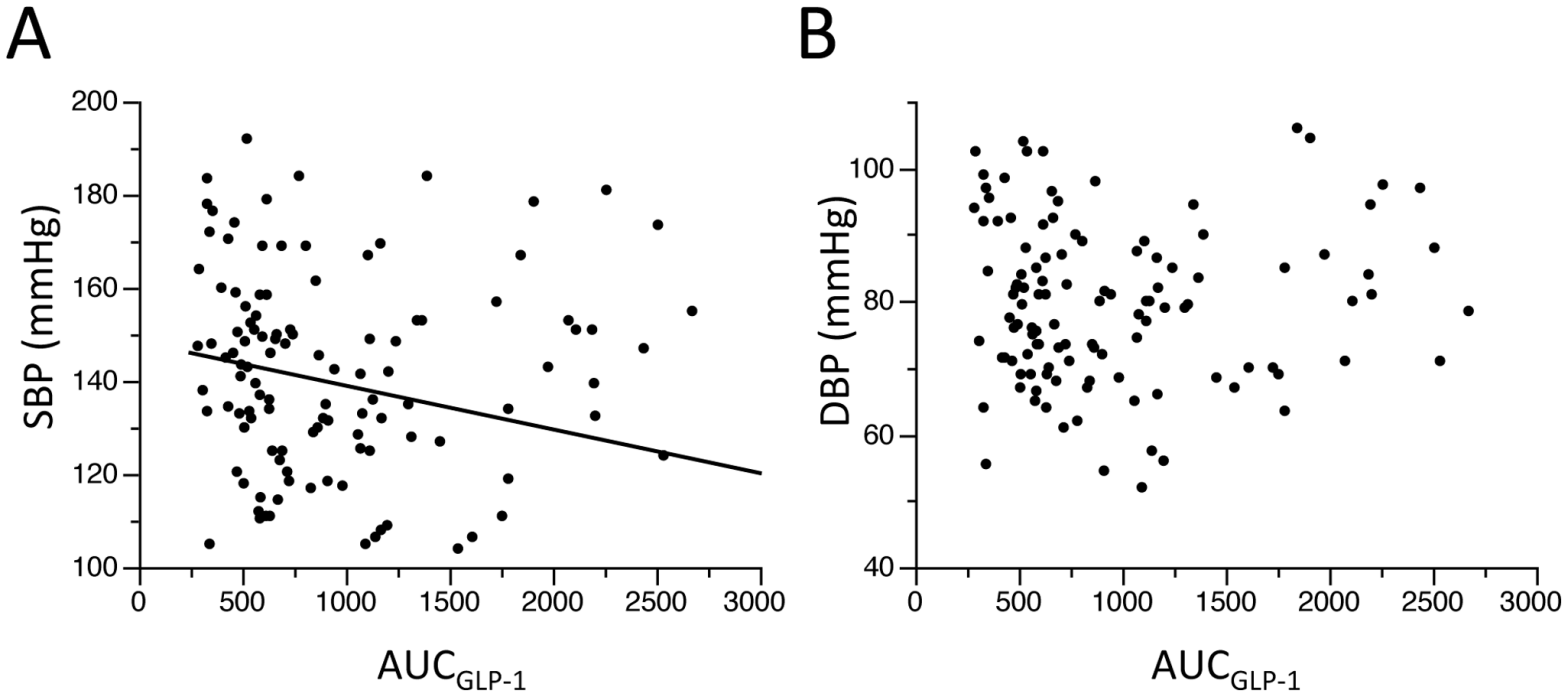
Relationship between blood pressure and AUC_GLP-1_. AUC_GLP-1_ was weakly correlated with SBP (r = −0.26, p = 0.0085, Panel A). However, a significant correlation was not observed for the AUC_GLP-1_– DBP relationship (Panel B). SBP, systolic blood pressure; DBP, diastolic blood pressure; AUC_GLP-1_, area under the curve of GLP-1 level in the oral glucose tolerance test.

Fasting GLP-1 level was not correlated with SBP or DBP (Table 2). In contrast, AUC_GLP-1_ was inversely correlated with SBP (r = −0.26, p = 0.0085) as shown in Figure 1, though such a correlation was not detected for DBP. Table 4 and Table 5 present results of multiple linear regression analyses for SBP and DBP with clinical variables. AUC_GLP-1_ and age were selected as independent determinants of SBP, though sex was the only variable associated with DBP in the present dataset. Replacement of fasting IRI with AUC_IRI_ in Tables 4 and 5 did not improve R^2^ values in regression for SBP or DBP (R^2^ data not shown).

**Table 4:** Multiple linear regression analysis for SBP and independent variables.

	B	SE	β	t	p
Sex	−3.26	2.01	−0.164	−1.62	0.108
Age (years)	0.500	0.240	0.233	2.08	0.040
BMI (kg/m^2^)	0.0383	0.568	0.00738	0.067	0.946
AUC_GLP-1_ (a.u.)	−0.00868	0.00366	−0.239	−2.37	0.020
Fasting IRI (µU/ml)	0.518	0.468	0.120	1.11	0.270
eGFR (ml/min/1.73 m^2^)	0.0124	0.166	0.00801	0.074	0.941

B, partial regression coefficient; SE, standard error; β, standardized partial regression coefficient; a.u., arbitrary unit.

The square of the coefficient of multiple correlation (R^2^) in this model = 0.17.

**Table 5:** Multiple linear regression analysis for DBP and independent variables.

	B	SE	β	t	p
Sex	−3.20	1.19	−0.279	−2.70	0.008
Age (years)	−0.0644	0.142	−0.0523	−0.455	0.650
BMI (kg/m^2^)	0.194	0.335	0.0652	0.579	0.564
AUC_GLP-1_ (a.u.)	−0.000767	0.00216	−0.0368	−0.355	0.723
Fasting IRI (µU/ml)	0.242	0.276	0.0972	0.876	0.383
eGFR (ml/min/1.73 m^2^)	0.0309	0.0982	0.0348	0.315	0.754

B, partial regression coefficient; SE, standard error; β, standardized partial regression coefficient; a.u., arbitrary unit.

The square of the coefficient of multiple correlation (R^2^) in this model = 0.12.

### Subgroup Analyses

**Figure 2:**
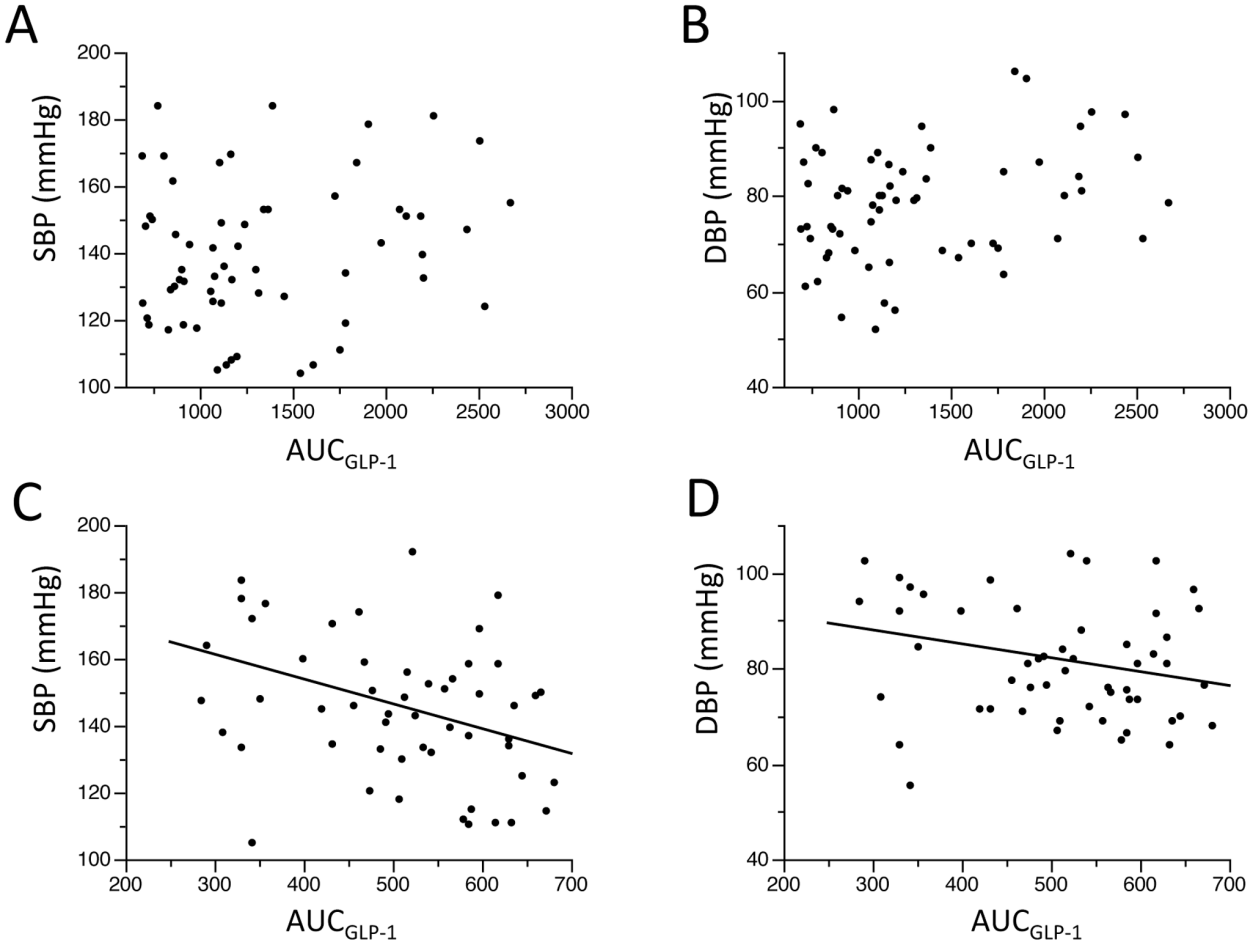
Blood pressure - AUC_GLP-1_ relationships in the high and low AUC_GLP-1_ groups. In the high AUC_GLP-1_ group, neither SBP nor DBP correlated with AUC_GLP-1_ (Panels A and B). In contrast, there were significant correlations between AUC_GLP-1_ and SBP (r = −0.40, p = 0.0032, Panel C) and between AUC_GLP-1_ and DBP (r = −0.28 p = 0.0448, Panel D) in the low AUC_GLP-1_ group. SBP, systolic blood pressure; DBP, diastolic blood pressure; AUC_GLP-1_, area under the curve of GLP-1 level in the oral glucose tolerance test.

To examine possible differences in demographic features between the subjects with high AUC_GLP-1_ and those with low AUC_GLP-1_, we divided the study subjects into two groups by the median of AUC_GLP-1_. As shown in Table 6, the low AUC_GLP-1_ group was younger, included a larger proportion of men and had lower baseline GLP-1 level, larger BMI and higher levels of LDL-C and eGFR than those in the high AUC_GLP-1_ group. HbA1c and indices of insulin sensitivities (i.e., Matsuda-DeFronzo index and HOMA-IR) were not significantly different between the two groups, though both indices tended to indicate lower insulin sensitivity in the low AUC_GLP-1_ group. Interestingly, both SBP and DBP were significantly correlated with AUC_GLP-1_ in the low AUC_GLP-1_ group (Figure 2), whereas such a correlation was not detected in the high AUC_GLP-1_ group.

**Table 6:** Demographic and clinical parameters in low AUC_GLP-1_ group and high AUC_GLP-1_ group.

	Low AUC_GLP-1_ Group (n = 51)	High AUC_GLP-1_ Group (n = 52)
Men, n (%)	30 (59)*	13 (25)
Age (year)	63±10*	67±8
BMI (kg/m^2^)	25.5±3.9*	22.6±3.1
SBP (mmHg)	146±20*	134±18
DBP (mmHg)	82±12*	76±11
Fasting PG (mg/dl)	97.1±9.9	96.0±9.6
AUC_PG_ (a.u.)	1736±359*	1561±286
Fasting IRI (µIU/ml)	7.0±5.6	5.5±3.0
AUC_IRI_ (a.u.)	616±450	542±338
Fasting GLP-1 (pmol/l)	2.75±0.76*	3.89±2.32
AUC_GLP-1_ (a.u.)	512±112*	1265±541
LDL-C (mg/dl)	139±28*	123±29
HDL-C (mg/dl)	62±15	69±27
Triglyceride (mg/dl)	118±55	117±70
HbA1c (%)	5.7±0.2	5.7±0.2
S-Cr (mg/dl)	0.72±0.15	0.70±0.14
eGFR (mL/min/1.73 m^2^)	75.3±13.7*	69.2±10.5
U-Alb/U-Cr (mg/gCr)	25.6±77.0	8.81±7.2
BNP (pg/ml)	17.8±12.3	23.9±19.4
hs-CRP (mg/dl)	0.10±0.11	0.07±0.09
Matsuda-DeFronzo index	7.9±5.2	8.3±3.9
HOMA-IR	1.7±1.5	1.3±0.8
HOMA-β	74.2±52.3	60.9±31.3

Data are presented as mean ± SD.

* p<0.05 vs. High AUC_GLP-1_ Group.

SBP, systolic blood pressure; DBP, diastolic blood pressure; PG, plasma glucose; IRI, immunoreactive insulin; AUC_GLP-1_, area under the curve of GLP-1 level during 75 g oral glucose tolerance test; S-Cr, serum creatinine; U-Alb/U-Cr, urinary albumin concentration-to-urinary creatinine concentration ratio; hs-CRP, high-sensitivity C-reactive protein; a.u., arbitrary unit.

**Figure 3:**
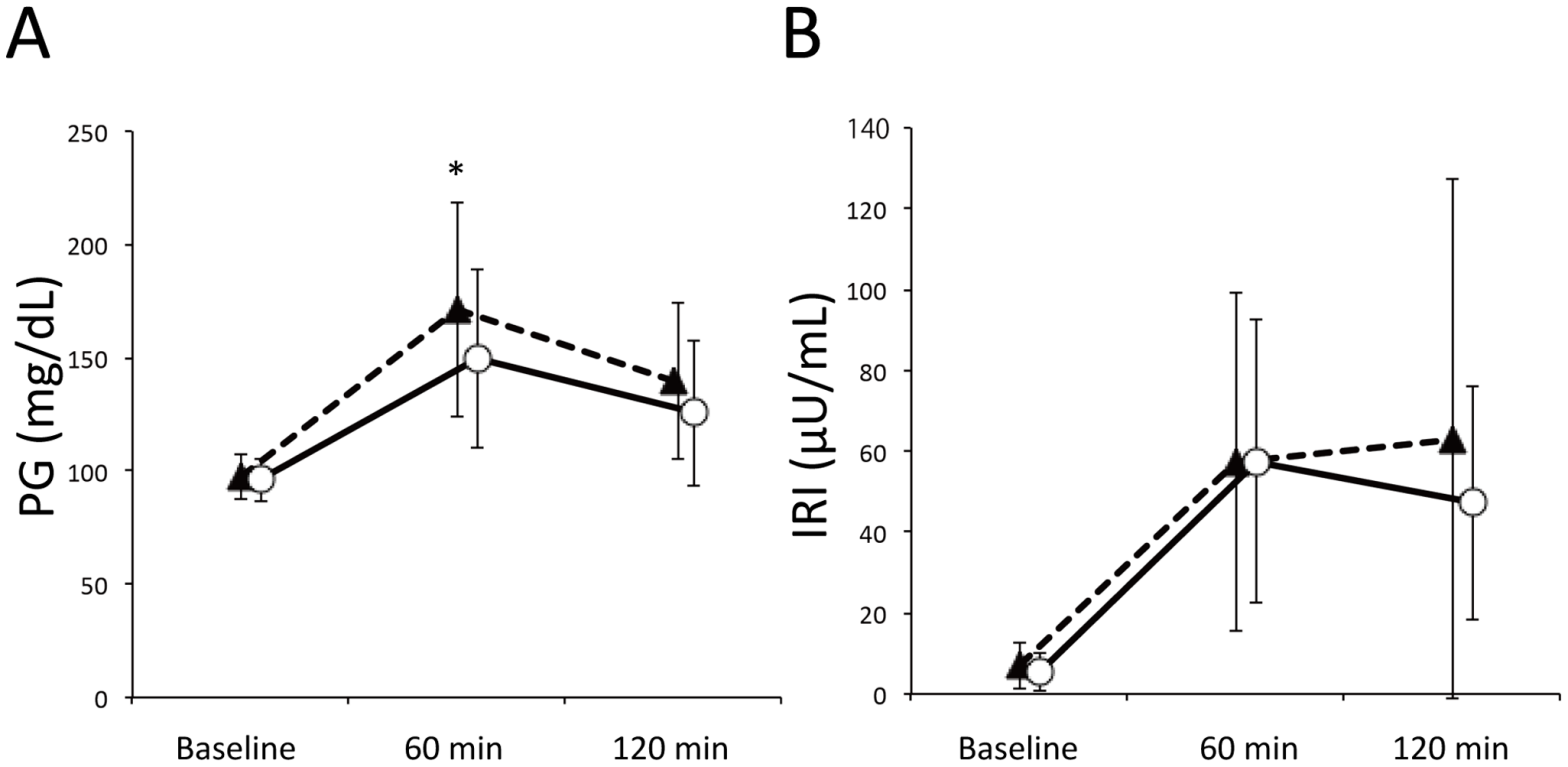
Time courses of PG and IRI in the OGTT. Broken lines and solid lines indicate the low AUC_GLP-1_ group and high AUC_GLP-1_ group, respectively. The low AUC_GLP-1_ group showed significantly higher PG level at 60 min after glucose loading than in the high AUC_GLP-1_ group (Panel A). *p = 0.039. There was no inter-group difference in time courses of plasma IRI during the OGTT (Panel B). PG, plasma glucose; IRI, immunoreactive insulin.

Time courses of PG and IRI levels during OGTT are shown in Figure 3. Levels of fasting PG were similar in the two groups (97.1±9.9 vs. 96.0±9.6 mg/dl). However, PG level at 60 min after oral glucose loading was significantly higher in the low AUC_GLP-1_ group than in the high AUC_GLP-1_ group (170.9±47.2 vs. 149.3±39.5 mg/dl). In contrast, IRI levels were similar before and after glucose loading in the high and low AUC_GLP-1_ groups. AUC_PG_ was significantly larger in the low AUC_GLP-1_ group than in the high AUC_GLP-1_ group, though AUC_IRI_ values were similar in the two groups (Table 6).

## Discussion

### Determinants of Baseline Plasma GLP-1 Level and GLP-1 Secretory Function

Since GLP-1 has extra-pancreatic targets of actions [1], [2], not only post-prandial but also baseline (fasting) level of GLP-1 potentially influence functions of extra-pancreatic tissues. However, a few studies have provided information on factors regulating baseline plasma GLP-1 level. Jørgensen et al. [17] reported that growth hormone deficiency induced an increase in fasting GLP-1 level. Pannacciulli et al. [18] showed that fasting GLP-1 level was positively correlated with rates of energy expenditure and negatively correlated with respiratory quotient, indicating a possible link between fatty acid oxidation and GLP-1 level. Since the level of daily energy expenditure potentially modifies insulin sensitivity, we assumed that insulin sensitivity indexes or lipid levels correlate with GLP-1 level. However, we did not find a significant correlation of baseline GLP-1 level with Matsuda-DeFronzo index, HOMA-IR, serum TG, or HDL-C. Also, no significant correlation was found between baseline GLP-1 and BMI, being consistent with results of earlier studies showing that fasting GLP-1 level in obese subjects was unchanged after weight reduction [19], [20]. In contrast to fasting GLP-1 level, post-prandial GLP-1 level was reduced after weight loss in a study by Adam et al. [19]. Nevertheless, the present observations indicate that insulin sensitivity or BMI are not contributory to physiological regulation of fasting GLP-1 level.

To our knowledge, there has been no study that systematically examined determinants of GLP-1 secretory function in non-diabetic subjects. Using AUC_GLP-1_ during OGTT, we examined the associations of GLP-1 secretory function with metabolic parameters and found that sex and age are independent determinants of AUC_GLP-1_ (Table 3). The results indicate that response of GLP-1 secretion to glucose loading is larger in women than in men. However, in an earlier study by Carroll et al. [21], time courses of GLP-1 during a 60-min postprandial period were similar in men and women. The number of subjects in their study was small (19 men and 20 women) and they used a 510 kcal test meal to evoke responses of GLP-1 and other hormones. Thus, absence of a sex difference in GLP-1 secretory function in the study by Carroll et al. [21] might have been a type II error or the sex difference in GLP-1 response in the present study might be specific to the situation of loading glucose only. This issue needs further investigation.

In contrast to our expectation, age was indicated to be a positive predictor of AUC_GLP-1_ in the present analysis (Table 3). We have no clear explanation for the mechanism underlying the positive age-AUC_GLP-1_ relationship. Since neither serum Cr nor eGFR was selected as an independent determinant of AUC_GLP-1_ in multiple linear regression analysis (Table 3), age-related decline in renal clearance of GLP-1 cannot explain the age-AUC_GLP-1_ relationship. One possible explanation is slower degradation of GLP-1 by DPP-4 in elderly subjects. However, this possibility is not supported by recent observations [22]–[24]. Korosi et al. [22] and Tahara et al. [23] showed that plasma DPP-4 activities were similar in young and elderly subjects. Damholt et al. [24] reported that plasma half-life of intravenously injected liraglutide, a GLP-1 analogue, was similar in young and elderly subjects, indicating comparable levels of DPP-4 activity in the two age groups. Another possible explanation for higher AUC_GLP-1_ in elderly subjects is the age-dependent increase in sensitivity of L-cells to glucose. However, that possibility is counterintuitive and currently lacks any supporting evidence.

### GLP-1 Secretory Function and PG Level after Glucose Loading

We could not find a significant relationship between AUC_GLP-1_ and HbA1c in the present study subjects, 32% of whom showed impaired glucose tolerance. However, as shown in Figure 3, PG levels after glucose loading were higher in the low AUC_GLP-1_ group than in the high AUC_GLP-1_ groups. On the other hand, time courses of IRI during the OGTT were similar in the two groups, indicating that insulin sensitivity was reduced in association with decrease in GLP-1 secretory capacity. Two indices of insulin sensitivity (Matsuda-DeFronzo index and HOMA-IR) tended to show lower insulin sensitivity in the low AUC_GLP-1_ group (Table 6), though the differences did not reach statistical significance. Furthermore, an association of insulin resistance and reduced response of plasma GLP-1 level to glucose loading has been observed in subjects with type 2 diabetes as well [3], [4]. Taken together, the present results indicate that reduced GLP-1 secretory function is associated with insulin resistance and post-prandial hyperglycemia even before insulin secretion is compromised.

The reason for the association between reduced GLP-1 secretion and insulin resistance is unclear. It is unlikely that change in plasma somatostatin level is involved in the association. Somatostatin, which suppresses GLP-1 secretion, was reported to be reduced in obese subjects [25] and in a rat model of metabolic syndrome [26]. In contrast, insulin has been shown to enhance GLP-1 secretion in an ERK-dependent manner, and this response of GLP-1 to insulin was attenuated by chronic hyperinsulinemia in human NCI-H716L cells and mouse models in vitro and in vivo [27]. Thus, there is the possibility that chronic hyperinsulinemia mediates association of insulin resistance and reduction in GLP-1 secretion in diabetic patients. However, in the present study subjects, in whom fasting IRI levels were mostly within the normal range, a significant correlation between fasting IRI and AUC_GLP-1_ was not detected (Table 2).

### Relationship between Endogenous GLP-1 and BP

GLP-1 has extra-pancreatic actions relevant to BP regulation [1], [2], [28]–[30]. The GLP-1 receptor has been shown to localize in endothelial cells and vascular smooth muscle cells [1], [2], and activation of the GLP-1 receptor induces nitric oxide production in endothelial cells and suppresses proliferation of vascular smooth muscle cells [28]–[30]. GLP-1 is also involved in sodium handling in the kidney. Gutzwiller et al. [5], [6] showed that GLP-1 increased sodium excretion in the proximal renal tubule in both healthy subjects and obese insulin-resistant subjects. Such a natriuretic action of a GLP-1 receptor agonist was not detected in GLP-1 receptor knockout mice [31]. BP tended to be higher in the GLP-1 receptor knockout mice than in wild-type mice, though the difference was not statistically significant. Furthermore, a recent study by Kim et al. [32] showed that activation of the GLP-1 receptor in the atria promotes secretion of atrial natriuretic peptide, leading to blood pressure reduction in mice. Collectively, the findings support the notion that the GLP-1 receptor in the vasculature, kidney and atria participates in BP regulation.

Consistent with the vasoprotective actions of GLP-1, GLP-1 infusion has been reported to improve flow-mediated vasodilatation in patients with type 2 diabetes [33]. In addition, Tesauro et al. [34] recently reported that loss of insulin-mediated enhancement of endothelial-dependent and -independent vasodilation in patients with metabolic syndrome was restored by infusion of GLP-1. This effect of GLP-1 was mimicked by vitamin C and the combination of GLP-1 and vitamin C did not further improve vasodilatory response, suggesting that the pathologic mechanism of reactive oxygen species production is a target of GLP-1. Furthermore, GLP-1 analogues and DPP-4 inhibitors have been shown to reduce BP (prior to reduction of body weight) in diabetic and non-diabetic patients with hypertension, [2], [8]–[11], [35]. In contrast to hypertensive subjects, normotensive subjects have been reported to be insensitive to the BP-lowering effect of GLP-1 [33], [36], [37]. GLP-1 infusion did not significantly change BP or heart rate unless hypoglycemia was induced [33], [36], [37]. However, the present study showed that an index of GLP-1 secretory function, AUC_GLP-1_, negatively correlated with SBP (Figure 1) and the association of AUC_GLP-1_ with SBP was independent of BMI, plasma IRI and age (Table 3 and Table 4). Furthermore, the relationship between BP and AUC_GLP-1_ was clearer in the group with low AUC_GLP-1_ (Figure 2). Taken together, the findings suggest that a slight decline in GLP-1 secretory function allows BP to elevate. In other words, preserved GLP-1 secretory function, leading to physiological GLP-1-mediated vasodilatation and natriuresis, may play a role in prevention of BP elevation.

Acute administration of GLP-1 or a GLP-1 analogue, exenatide, does not decrease BP in healthy subjects [36], [38]. Thus, the inverse correlation between AUC_GLP-1_ and SBP (Figures 1 and 2) is unlikely to be mediated by acute effects of GLP-1 on the vasculature and/or the sympathetic nervous system and perhaps reflects chronic vasoprotective and natriuretic actions of endogenous GLP-1 [39]–[41].

Level of daily sodium intake is an established determinant of BP, and our recent study confirmed that estimated sodium intake correlated with BP in subjects in the Tanno-Sobetsu cohort [42]. Sodium intake levels estimated from sex, body weight, urinary sodium and Cr data by use of the equation for Japanese [43] were 13.6±3.8 g/day in men and 12.5±3.5 g/day in women in this cohort. It would have been interesting if natriuresis could have been assessed for examination of the relationship between level of natriuresis and AUC_GLP-1_. However, it is not clear whether estimated sodium intake is sensitive enough for analysis of a modest change in natriuresis by its regulatory factors, and collection of 24-h urine samples from a general population is difficult. Thus, we did not attempt to directly examine relationship between natriuresis and AUC_GLP-1_ in the present study.

### Limitations in the Present Study

There are several limitations in this study. First, subjects in the Tanno-Sobetsu cohort received OGTT on a voluntary basis in addition to examinations mandatory for registration, and invitation to undergo an OGTT was based on FPG and HbA1c data indicating possible glucose intolerance. Thus, selection bias for both higher health-oriented subjects and those with lower glucose tolerance is likely to be present in this study. Second, we excluded all subjects on regular medications and aimed to exclude patients with untreated diabetes and cardiac diseases primarily by medical history (i.e., questionnaire). Thus, the possibility of asymptomatic and undiagnosed cardiovascular diseases could not be ruled out. In fact, BP was above normal limits in approximately half of the study subjects, though diagnosis of hypertension cannot be made by a single measurement of BP. Third, the present study has limitations due to observational cross-sectional analysis and we could not critically discuss cause-and-results relationships for significant associations of AUC_GLP-1_ with age and with BP. Longitudinal analyses are necessary to confirm the present findings, particularly age-related change in GLP-1 secretory function and relationship between BP and GLP-1 secretory function.

### Conclusion

Multiple linear regression analyses of data from subjects on no medication in a general population indicated that an index of GLP-1 secretory function, AUC_GLP-1_, positively correlated with age and negatively correlated with SBP. Endogenous GLP-1 may be involved in BP regulation, and age-dependent increase in GLP-1 secretory response to glucose loading might be an adaptive response. Mechanisms underlying the changes in AUC_GLP-1_ and the relationship between endogenous GLP-1 and BP regulation remain to be further studied.
